# Health-Related Quality of Life Issues Experienced by Thoracic and Breast Sarcoma Patients: A Rare and Understudied Group

**DOI:** 10.3390/jcm10225334

**Published:** 2021-11-16

**Authors:** Ilse van Eck, Dide den Hollander, Emma Lidington, Leopold Hentschel, Martin Eichler, Samer Salah, Susanne Singer, Monica Pinto, Lena Fauske, Marco Fiore, Ioanna Nixon, Anastasia Constantinidou, Ingrid M. E. Desar, Johannes J. Bonenkamp, Winan J. van Houdt, Milou J. P. Reuvers, Rick L. M. Haas, Øyvind S. Bruland, Bernd Kasper, Winette T. A. van der Graaf, Olga Husson

**Affiliations:** 1 Department of Medical Oncology, The Netherlands Cancer Institute, Antoni van Leeuwenhoek, 1066 CX Amsterdam, The Netherlands; i.vaneck@student.ru.nl (I.v.E.); D.denhollander@radboudumc.nl (D.d.H.); m.reuvers@nki.nl (M.J.P.R.); w.vd.graaf@nki.nl (W.T.A.v.d.G.); 2 Department of Medical Oncology, Radboud University Medical Center, 6525 GA Nijmegen, The Netherlands; Ingrid.Desar@radboudumc.nl; 3 The Royal Marsden NHS Foundation Trust, London SW3 6JJ, UK; emma.lidington@rmh.nhs.uk; 4 National Center for Tumor Diseases (NCT/UCC), University Hospital Carl Gustav Carus, Technical University Dresden, 01307 Dresden, Germany; Leopold.Hentschel@uniklinikum-dresden.de (L.H.); Martin.Eichler@uniklinikum-dresden.de (M.E.); 5 Department of Medical Oncology, King Hussein Cancer Center, Amman 11941, Jordan; DS.06907@KHCC.JO; 6 Institute of Medical Biostatistics, Epidemiology and Informatics, University Medical Centre Mainz, 55131 Mainz, Germany; singers@uni-mainz.de; 7 University Cancer Centre Mainz (UCT), University Medical Centre Mainz, 55131 Mainz, Germany; 8 Rehabilitation Unit, Department of Supportive Care, Instituto Nazionale Tumori-IRCCS-Fondazione G. Pascale, 80131 Naples, Italy; monicapinto@iol.it; 9 Department of Oncology, The Norwegian Radium Hospital, Oslo University Hospital, 0379 Oslo, Norway; lenfau@ous-hf.no (L.F.); osb@ous-hf.no (Ø.S.B.); 10 Department of Interdisciplinary Health Sciences, Institute of Health and Society, University of Oslo, Blindern, 0317 Oslo, Norway; 11 Department of Surgery, Fondazione IRCCS Istituto Nazionale dei Tumori, 20133 Milan, Italy; Marco.Fiore@istitutotumori.mi.it; 12 Department of Clinical Oncology, Beatson West of Scotland Cancer Centre, Glasgow G12 0YN, UK; ioanna.nixon@ggc.scot.nhs.uk; 13 Medical School University of Cyprus, Bank of Cyprus Oncology Centre, 1678 Nicosia, Cyprus; constantinidou.anastasia@ucy.ac.cy; 14 Department of Surgical Oncology, Radboud University Medical Center, 6525 GA Nijmegen, The Netherlands; han.bonenkamp@radboudumc.nl; 15 Department of Surgical Oncology, The Netherlands Cancer Institute, Antoni van Leeuwenhoek Hospital, 1066 CX Amsterdam, The Netherlands; w.v.houdt@nki.nl; 16 Department of Radiotherapy, The Netherlands Cancer Institute, Antoni van Leeuwenhoek Hospital, 1066 CX Amsterdam, The Netherlands; r.haas@nki.nl; 17 Institute of Clinical Medicine, University of Oslo, Blindern, 0316 Oslo, Norway; 18 Sarcoma Unit, Mannheim University Medical Center, University of Heidelberg, 68167 Mannheim, Germany; bernd.kasper@umm.de; 19 Department of Medical Oncology, Erasmus MC Cancer Institute, Erasmus University Medical Center, 3015 GD Rotterdam, The Netherlands; 20 Division of Clinical Studies, Institute of Cancer Research, London SM2 5NG, UK

**Keywords:** breast sarcoma, thoracic sarcoma, chest sarcoma, health-related quality of life, patient-reported outcomes

## Abstract

Thoracic and breast sarcomas constitute a rare subgroup within the sarcoma population. There is limited knowledge about their health-related quality of life (HRQoL) and a valid disease-specific HRQoL instrument is lacking. This qualitative study aimed to investigate the HRQoL issues experienced by a small group of thoracic and breast sarcoma patients. Semi-structured interviews with 19 thoracic and four breast sarcoma patients were conducted and thematically analysed. Physical issues mentioned by both groups were fatigue, sleep disturbances, pain, wound infections, and symptoms related to chemotherapy and radiotherapy. Tightness in the back and restrictions in performing tasks above arm height were specific physical issues for breast sarcoma patients, whereas respiratory problems were only mentioned by thoracic sarcoma patients. Body image issues, changes in mood, fear of recurrence, and living with uncertainty were important mental health issues for both subgroups. Social issues in both groups included challenges in work and relationships, financial difficulties, loss of independence, and limitations in social activities. The identified physical, mental, and social health challenges can significantly impact thoracic and breast sarcoma patients’ HRQoL. Results of this qualitative study will guide personalised supportive care for breast and thoracic sarcoma patients and help in determining the best possible HRQoL measurement strategy for sarcoma patients with different primary sarcoma locations.

## 1. Introduction

Sarcomas represent a rare group of cancers that account for only 1% of all adult malignancies [1]. They are very heterogeneous, covering more than 100 histological subtypes, which can develop anywhere in the body [1,2]. The treatment of sarcomas is often accompanied by significant short- and long-term side effects that negatively affect patients functioning in daily life. Focusing on patient-reported outcomes (PROs), such as health-related quality of life (HRQoL), is therefore important. In the study of Eichler et al. [3], the heterogeneity of sarcomas with regard to type and location was reflected in HRQoL outcomes. Additionally, in our previous work, in which we used the European Organisation for Research and Treatment of Cancer Quality of Life Questionnaire (EORTC QLQ-C30) as a measure of HRQoL in combination with unique treatment-specific HRQoL questions for each sarcoma location [4], we found different patterns of HRQoL per sarcoma location, but also additional treatment-specific HRQoL issues which seemed unique for each tumour site. For example, difficulty seeing themselves naked after surgery was specifically mentioned by breast sarcoma patients. These additional treatment-specific HRQoL issues are not captured by generic or cancer-generic HRQoL questionnaires. Hence, these instruments are not specific enough to capture all issues relevant to patients with different sarcoma locations and treatments and lack content validity [5,6].

To overcome this problem, a comprehensive HRQoL measurement strategy is currently being investigated for sarcoma patients. The EORTC quality of life group and EORTC soft tissue and bone sarcoma group will determine which strategy should be adopted for HRQoL measurement in patients with different sarcoma locations [7]. In order to determine the best measurement strategy, it is important to gain insight into the unique issues by sarcoma site. Two, especially underrepresented, location-specific subgroups of sarcomas in HRQoL research are primary thoracic (also referred to as chest sarcomas) and primary breast sarcomas [8].

Thoracic sarcomas represent approximately 5% of all thoracic neoplasms [9]. They can arise from all anatomic structures of the thorax, including the lung, mediastinum, pleura, and chest wall (from both soft tissue and bone). Clinical features depend on the anatomical location and histology of the sarcoma. For example, leiomyosarcomas located in the mediastinum typically manifest with local mass effect (e.g., pain, cough, superior vena cava syndrome), whereas patients with pulmonary and bronchial rhabdomyosarcomas can present with cough, dyspnoea, haemoptysis, and pneumothorax [10]. The primary treatment is surgical resection with uninvolved histological margins, usually pre-treated with or followed by radiotherapy and/or to a lesser extend adjuvant chemotherapy depending on the histology of the tumour and patient characteristics [10,11]. For locally advanced tumours infiltrating in critical anatomic structures, surgical resection can lead to high morbidity and full-thickness defects of the thoracic wall [12]. Reconstructing the thoracic wall through various medical products, tissue transfer, and plastic surgery techniques can reduce surgical morbidity and provide sufficient stability to maintain pulmonary function [12]. Nevertheless, patients with thoracic wall reconstruction still deal with limitations in breathing, postoperative pain, sensation disorders, or limitations in daily activities [13].

Breast sarcomas account for less than 5% of all soft tissue sarcomas and less than 1% of all breast malignancies [14]. Based on aetiology, they can be divided into two categories: de novo sarcomas (primary sarcomas) and radiation-induced sarcoma of the breast. Radiation-induced sarcoma of the breast is associated with worse outcomes, compared with primary breast sarcomas [15,16]. Primary breast sarcomas can present as a haematoma-like skin discoloration (angiosarcoma) or as a palpable mass, similar to breast carcinomas but needing different diagnostic and treatment strategies. They also have a high risk of recurrence and a significantly worse prognosis [14,17]. Breast carcinomas are often treated with breast conservative surgery (BCS) combined with (neo)adjuvant chemotherapy and/or adjuvant radiotherapy. For breast sarcomas, the preferred treatment is complete resection with wide margins, usually by mastectomy. In comparison, treatment for breast carcinomas can lead to negative changes in identity [18], self-esteem [19], and body image [20] issues which breast sarcoma patients may also experience but have not been investigated yet.

Considering the aforementioned impact of treatment in the thoracic and breast area, and the limited research about HRQoL issues in both location-specific subgroups of sarcomas, the aim of this study was to assess the unique issues experienced by patients with primary thoracic and primary breast sarcomas. As the location of these two types of sarcomas are closely related and it is assumed that the impact of the treatment will overlap, they are both described in this article.

## 2. Materials and Methods

### 2.1. Sample and Procedure

This paper describes a predefined subgroup analysis of data collected within a large multicentre phase 1 EORTC study [7]. For this subgroup analysis, only sarcoma patients aged ≥ 18 years diagnosed with thoracic and breast sarcomas (according to the ICD-10 codes; C34 (malignant neoplasm of bronchus and lung), C38 (malignant neoplasm of heart, mediastinum, and pleura), C49.3 (malignant neoplasm of the connective and soft tissue of thorax), C41.3 (malignant neoplasm of ribs, sternum, and clavicle), and C50 (malignant neoplasms of the breast)) were included. Although sarcoma is a disease affecting all ages, and sarcoma patients aged < 18 years are seen within adult health services in some countries, only those aged 18 years and older were included because the EORTC QLQ-C30 is only validated among cancer patients aged 18 years and older. Both patients currently undergoing treatment and patients in the follow-up period were included and asked to describe their sarcoma journey and/or follow-up trajectory. Participants in this analysis were from Germany, Norway, The Netherlands, Italy, Cyprus, and the United Kingdom. Purposive sampling was used to obtain a varied sample with regard to age, sex, treatment, tumour location, subtype, and stage. Patients with cognitive impairment or a physical condition hampering adequate participation (judged by their (ex-) treating physician) were excluded. In addition, patients with gastrointestinal stromal tumours (GIST), Kaposi sarcoma, and carcinosarcoma were excluded, because of the unique clinical behaviour of these sarcomas in terms of the type of disease and treatment. Two thoracic sarcoma patients with a dermatofibrosarcoma of the skin of the thorax were excluded, as treatment and prognosis are considerably different for this subtype.

The clinical team initially approached potential participants. If he or she expressed interest, a member of the research team further explained the study and participation. All participants gave written informed consent prior to the interview. Ethical approval was provided by the Institutional Review Board of The Netherlands Cancer Institute (IRB18121) as the institution’s medical ethics committee considered the study to be outside the Dutch Medical Research Involving Human Subjects Act (WMO). The study was also reviewed and approved by the national research regulatory authorities and local medical ethical committees for each participating country and institute according to local regulations.

### 2.2. Interviews

Interviews were conducted by local collaborators in the native language of the patient following a semi-structured interview schedule [7]. Prior to the interview, patients answered a short questionnaire about sociodemographic characteristics.

### 2.3. Data Analysis

With permission, all interviews were recorded and transcribed smoothly verbatim. Data analysis was conducted by three coders (M.J.P.R., D.d.H., I.v.E.) via Nvivo 12 using thematic analysis [21,22]. To become familiar with the interview coders repeatedly read the transcripts and, to reduce bias, independently highlighted sections in the transcripts that were related to the research objectives and coded these into key themes following a predefined model (the biopsychosocial model). Using this inductive way of analysis, separate HRQoL themes were identified for breast sarcomas as well as thoracic sarcomas. We excluded positive issues because they are not part of the construct of HRQoL [23]. The biopsychosocial model systematically considers biological, psychological, and social factors and their complex interactions in understanding health, illness, and health care delivery [24]. Within the three main themes, coders independently defined subthemes. Afterwards, coders discussed their findings, refined the subthemes, and resolved differences until consensus was reached. All quotes were anonymised (Tables S1–S6 in the Supplementary Materials).

## 3. Results

### 3.1. Participants 

In total, 19 patients with thoracic sarcoma and four patients with breast sarcoma were interviewed. Sociodemographic and clinical characteristics are presented in Table 1.

### 3.2. Interviews

**Figure 1:**
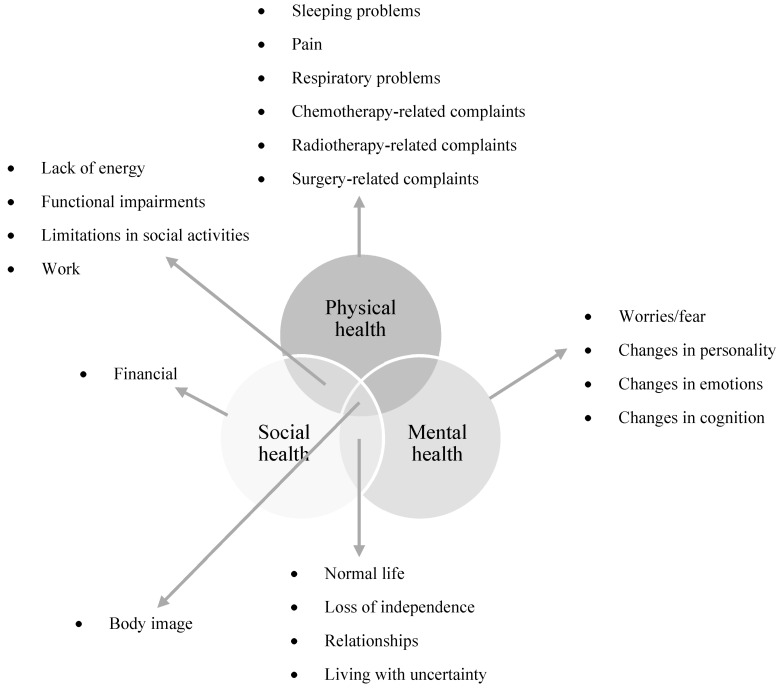

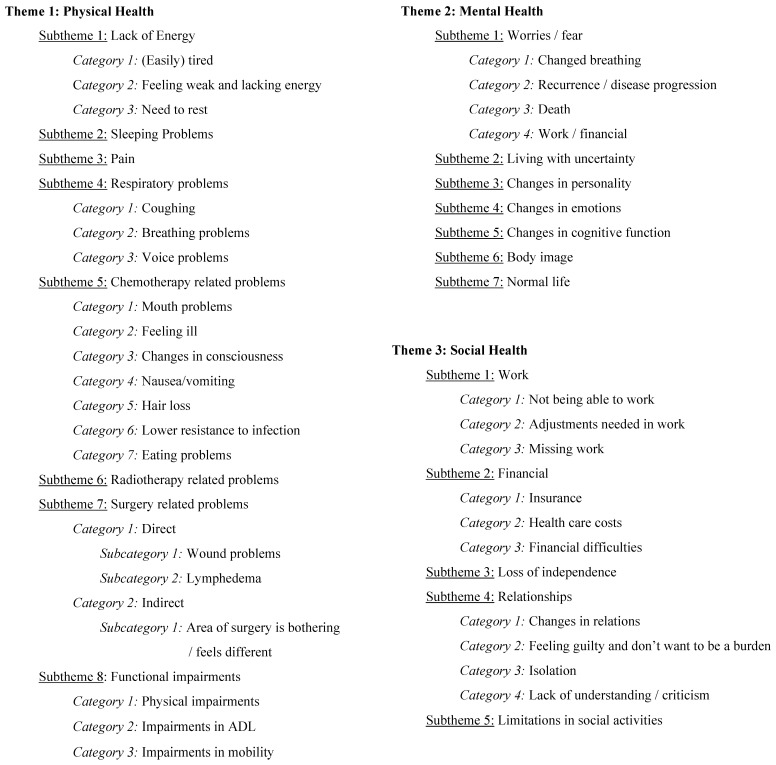
Three-dimensional theoretical framework of health for thoracic sarcoma patients and the different subthemes within the main themes physical, mental, and social health based on the biopsychosocial model.

**Figure 2:**
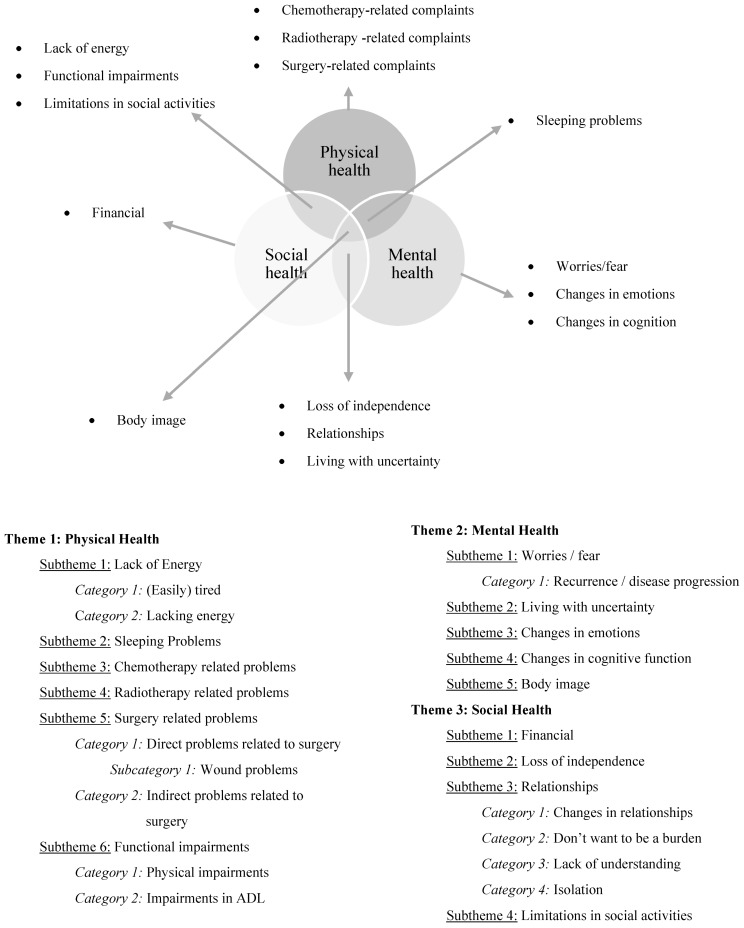
Three-dimensional theoretical framework of health for breast sarcoma patients and the different subthemes within the main themes physical, mental, and social health based on the biopsychosocial model.

Interviews were conducted between May 2019 and September 2020. The duration of the interviews ranged from 15 to 74 min. Within the main theme ‘physical health’, eight subthemes were defined for thoracic sarcomas and six for breast sarcomas. Within the main theme ‘mental health’, seven subthemes were defined for thoracic sarcomas and five for breast sarcomas, and within the main theme ‘social health’, five subthemes were defined for thoracic sarcomas and four for breast sarcomas (Figure 1 and Figure 2). Some of these subthemes were overlapping between both locations (e.g., changes in emotions), but other issues were only mentioned by one sarcoma subgroup. For example, respiratory problems were only mentioned by thoracic sarcoma patients. Therefore, this subtheme was only created for the thoracic sarcoma subgroup. In addition, some of these subthemes refer primarily to one of the three main themes (e.g., chemotherapy-related complaints primarily reflecting physical health), and others refer to two or three main themes (e.g., body image reflects physical, mental, and social health) (Figure 1 and Figure 2). Additionally, between patients with thoracic and breast sarcomas, the same subtheme can refer to different main themes (e.g., in patients with thoracic sarcomas, sleeping problems reflect physical health issues, while in patients with breast sarcomas, sleeping problems reflect both physical as well as mental health issues). The different subthemes were further divided into categories (Figure 1 and Figure 2). Issues associated with hormonal therapy which was given to one thoracic sarcoma patient in an experimental context were excluded since this treatment is not used in daily practise.

### 3.3. Physical Health 

After diagnosis and treatment, both breast and thoracic sarcoma patients felt tired in general or tired more quickly when performing activities. Furthermore, lack of energy was mentioned by patients with sarcoma of both locations, whereas thoracic sarcoma patients also mentioned weakness and the desire to rest more often. Tiredness and lack of energy were experienced during treatment: ‘I just wanted to sleep, because after the second chemo I had anaemia.’, but also in the follow-up period, causing a substantial impact on patients’ everyday life. Patients mentioned the need to rest after cooking, the need to take a nap sometimes, being partially disabled because of fatigue, and not having the same energy as before.

Sleeping problems were also mentioned by patients with sarcoma in both locations. Breast sarcoma patients experienced sleeping problems mostly due to worries at night, although one breast sarcoma patient mentioned having trouble sleeping due to limited positions to sleep in after surgery caused by transposition of back musculature to the front: ‘I could not really lie on my back and my side is also a bit difficult’. One thoracic patient mentioned sleeping problems because of her changed breathing: ‘It is as if I am not getting enough oxygen. I’m a little bit scared about that or panicky sometimes. It is annoying. Normally during the day, I have no problems with it, nor do I suffer from it. But when I am in bed, I am paying attention to it in a different way’.

Respiratory problems, including coughing, shortness of breath, problems with breathing, being out of breath often, and voice problems were only reported by thoracic sarcoma patients. Additionally, pain, especially after surgery, but also in the follow-up period, was common in thoracic sarcoma patients, causing difficulties with lifting heavy weights, problems with lying in a certain position, and difficulty performing normal daily activities.

In addition to fatigue, the extensive chemotherapy treatments caused a variety of complaints including mouth problems, eating problems, nausea/vomiting, hair loss, feeling ill, changes in consciousness, lower resistance to infection, headaches, heart palpitations, shortness of breath, urinary incontinence, regurgitation of food, a sagging feeling of the feet and losing nails. Patients felt well for a limited number of days: ‘And when I just recovered a bit, I had to go again and I found that very difficult’ and their activities were limited by these complaints: ‘I didn’t go to birthdays because it made me sick, because at a certain point my resistance became worse and worse’.

Radiotherapy caused skin problems in one thoracic sarcoma patient. Another thoracic patient mentioned irritation of the oesophagus, leading to problems with eating and the need for a feeding tube after radiotherapy. Changed breathing was described after radiation of the breast, which did not improve after ending the treatment.

The extensive surgery sometimes led to wound problems in both thoracic and breast sarcoma patients. Patients described wound closure problems, infection of the wound, reopening of the wound, the need to flush the wound, and the need for a vacuum pump. Lymphedema of the arm was mentioned as a direct consequence of surgery of the thorax as well. Indirect consequences mentioned after thoracic surgery were a different/bothering feeling in the operated area. One patient explained this feeling as: ‘imagine you’re wearing a three-quarter sleeve t-shirt from your six-year-old nephew. So every movement you do, it works, but it feels like it’s impossible’, and another patient felt the placed mat where they removed two ribs: ‘The doctor removed the bottom floating ribs and a mat has been placed in it. When I turn around, I can feel it stinging a little’. Location-specific indirect complaints mentioned after surgery of the breast included a tight feeling or shortness of breath, caused by transposition of back musculature to the front. One patient said: ‘sometimes I think, oh, I still have my bra on, but I’ve already taken it off. It feels that tight’. Another patient experiencing shortness of breath needed physiotherapy to learn how to bring the breathing back to normal.

Some physical problems caused by either the tumour itself or its treatment had a significant impact on the patients’ physical functioning. Patients described performing less activity than before the diagnosis due to a lack of energy and body strength. They had to adjust their degree and pace of physical activity and some tasks took longer than before. Commonly described physical impairments were restrictions in walking distance and physical exertion, such as running or climbing stairs. Some patients found the necessary adjustments to physical activity and difficulty finishing tasks they used to be able to complete annoying. Problems in activities of daily living (ADL) during and after treatment were described by patients of both locations as well, mainly physical constraints when doing (heavy) household chores or when performing tasks above arm height: ‘What I find most difficult is cleaning on top of cupboards or cleaning windows’.

### 3.4. Mental Health

Fear and anxiety about disease progression or recurrence of the disease was a major concern for both subgroups. An increase in tension was particularly mentioned for the days before follow-up scans. Other concerns thoracic sarcoma patients dealt with were fear about changed breathing, fear of death, fear of not seeing children grow up, and financial constraints.

Living with uncertainty is an important HRQoL issue mentioned by both thoracic and breast sarcoma patients. Patients were uncertain about the cause of their disease and the treatment trajectory they had to undergo, and they were aware that sarcomas often recur: ‘I never felt cured either, it is known that it is a tumour that can come back quickly.’ Not only the chance of recurrence but especially the risk of death made living with a sarcoma diagnosis emotionally difficult. One patient who had a history of breast cancer explained having more difficulty dealing with the sarcoma diagnosis than the breast cancer diagnosis: ‘With the breast cancer, I still had the false safety of well, it might go wrong, but it will take a while. I still have some time. And with this one it feels like it is panting in my neck. It really feels like a race against time, next month it could be all wrong and be done’.

Both subgroups experienced changes in mood and negative emotions due to their disease and treatment: feeling angry, sad, tense, down, worried, and depressed. In addition, changes in personality such as being aggressive and grumpy, more emotionally sensitive, and less self-confident were described. Changes in cognitive functioning such as memory loss and difficulties concentrating were mentioned after treatment, leading to problems especially in work and the need for adjustments in everyday life: ‘I have trouble remembering things. My house is full of notes’.

Being dependent on others was a major change for many patients. Partners, parents, children, or friends had to take over household chores or support the patient with transport. Some felt troubled about dependency on others and one patient found needing support with washing themself especially difficult: ‘I thought it was so bad that I was sitting naked on a bench in the shower, and other people had to shower me. I thought that was terrible’.

Negative body image and acceptance of the altered body is an (ongoing) challenge for patients with sarcoma of both locations due to scars after surgery and loss of hair after chemotherapy. In particular, patients who had a breast amputation had difficulties with seeing themselves naked; one patient called it ‘a mutilation of her body’. Negative body image affected patients’ social life, for example, patients described not going to the sauna to avoid other people staring at their scars or not going to a friend’s birthday or seeing their grandchildren because of hair loss. Patients tried to hide the scars with clothes by not wearing spaghetti straps in the summer and not wearing low necklines. Other patients accepted their changes in appearance more but found it hard to deal with the reactions of other people: ‘I remember that I had shown it to a friend who was shocked. I am very open. I take off my shirt and I show it. But it is also difficult how to deal with the reaction of others’.

Avoiding hair loss was important for a patients’ identity, but also having a good alternative when hair loss occurs (wig, cap, and hat) was helping in maintaining a positive body image. One patient mentioned not liking wigs, because *‘it looks ugly’* and searched for other alternatives. Keeping hair was also important for feeling normal: ‘It was very important for me that I could just walk down the street like nothing is wrong with me’.

Furthermore, being able to accomplish things in the same way as before the diagnosis was important for feeling ‘normal’. One patient still went to rehearsal every week, even though she became weaker and weaker: ‘I continued to do that, and I subconsciously think, I maybe did that, because I wanted to feel normal’. The desire to feel normal was a very important issue for many patients.

### 3.5. Social Health

The physical and emotional impact of a thoracic or breast sarcoma diagnosis and the effect of treatment on patients’ body image had a profound impact on social activities and well-being. The diagnosis caused disruption of various aspects of the patients’ social life, including work, financial situation, relationships, and social activities.

Some patients mentioned not being able to work anymore or being partially disabled, because of concentration problems, fatigue, or lack of strength. Patients needed adjustments in work: partially working from home so the patient could rest in between meetings, reducing the workload, needing help with some tasks, and not scheduling appointments that cannot be cancelled last minute.

Not being able to work was sometimes accompanied by financial difficulties: ‘I get financial support from my girlfriend, because I am disabled, so I can’t work anymore’. Other financial difficulties were caused by high health care costs: ‘a wig is very expensive’ and, specifically for breast sarcoma patients, high costs of new clothes that fit the breast prosthesis: ‘You have to buy a bathing suit and it immediately costs EUR 140 because you have to put a prosthetic in. You have to buy a prosthesis. I still wear normal bras, but actually you also have to buy new bras’. Additionally, obtaining a new mortgage became more difficult.

The side effects of treatment and long-term effects of the disease caused changes in relationships for several patients. Some patients mentioned they lost contact with people or saw friends less because of physical limitations, while others were not always able to participate in activities that friends suggested. Some patients felt isolated after their diagnosis or felt their world became smaller. Patients felt alone with their feelings because they did not want to burden their family or did not have a partner to share feelings with: ‘I can’t always get everything of my chest, because I don’t want to burden my children with it every time’. Others felt they were a burden to their family because family members changed their plans because of their illness: ‘So my son said, ‘You will probably never see us having children and if we get married now, you can still be there’. I said, ‘You completely ignore your own wishes’. Additionally, feelings of guilt for hurting their family and difficulty sharing bad news with family members was mentioned by both subgroups: ‘They start crying and ask why? Nobody knows why, so I felt guilty for hurting them’.

Another issue patients found difficult was that people could not empathise with how the sarcoma diagnosis influences their life. Different coping styles, instructions from others on how to deal with the disease, and unwanted well-intentioned advice caused tension and irritation, sometimes leading to less contact between patients and family. Some patients found it difficult to have to explain the situation to others repeatedly: ‘You are the one who has the disease and you are also the one who also has to tell, it’s not like this, it is like this. That also has an impact on yourself. That’s hard sometimes. You also need energy for that’.

Patients also experienced limitations in leisure activities. They could not always enjoy their hobbies as they used to, which they found frustrating or made them ‘*rebellious*’. One patient with a thoracic sarcoma could not play football anymore, because of the two missing ribs after surgery. In addition, social occasions, such as birthday parties, were not always feasible: ‘there is a lot of talking that makes me dizzy’. They had to plan social activities depending on the other activities they had to accomplish that day and sometimes had to cancel: ‘I cannot have a busy program in the morning, and have a busy program in the afternoon, and in the evening. So, it’s either one of them’. Social activities could no longer be spontaneous.

## 4. Discussion

In this study, thoracic and breast sarcoma patients mentioned cancer generic issues, as well as issues related to the location and specific treatment of the sarcoma subtype. Cancer generic issues mentioned by both groups were fatigue, sleep disturbances, pain, wound infections, and symptoms related to surgery, chemotherapy, and radiotherapy. Tightness in the back and restrictions in performing tasks above arm height were specific physical issues for breast sarcoma patients, whereas respiratory problems were only mentioned by thoracic sarcoma patients. Body image issues, changes in mood, fear of recurrence, and living with uncertainty were important mental health issues for both groups. Social issues in both groups included challenges in work and relationships, financial difficulties, loss of independence, and limitations in social activities.

Fatigue is one of the most common and distressing side effects of all types of cancer and its treatment [25]. Fatigue usually improves in the year after treatment; nevertheless, Bower et al. [26] found that a third of breast cancer patients still report significant fatigue at 5-10 years after diagnosis. Additionally, sleep disturbances are common in patients with all types of cancer and are frequently caused by worries, concerns, and pain or discomfort [27]. Thoracic and breast sarcoma patients in our study mentioned different causes of sleep disturbances (worries and limited positions to lie in after surgery in breast sarcoma vs. changes in breathing in thoracic sarcoma), indicating that it might be important to assess the cause of a patient’s sleep disturbance in order to adequately tackle the problem with pharmacological and/or psychological interventions. Furthermore, pain is a common issue in all types of cancer but also in the sarcoma population in general in which it is often treated inadequately [28]. In our previous study, we found that the type and location of pain were different between the different sarcoma locations and treatments; i.e., pain in muscles or joints after chemotherapy, phantom pain in extremity sarcoma patients who underwent an amputation, or pain in the affected breast after breast surgery [4], indicating that it is not only important to assess if the patient has pain, but it might also be important to assess the location, type, and frequency of the pain in order to accomplish pain relief and optimise HRQoL. The majority of complaints caused by chemotherapy and radiotherapy experienced by the thoracic and breast sarcoma patients in our study are also commonly experienced by patients with other types of cancer [29,30,31], except for the changed breathing after radiotherapy, which is a well-known location-specific adverse event after radiotherapy for tumours of the thorax including patients with carcinomas in the breast or lung [32,33].

Respiratory problems were location-specific problems for thoracic sarcoma patients only. Coughing and dyspnoea are common (long-term) complaints after treatment in lung cancer patients as well, leading to disrupted sleep, difficulties talking, reduced levels of physical functioning, limitations in physical activities, and decreased social functioning [34,35]. These limitations were also described by thoracic sarcoma patients in our study. Assessment of respiratory distress might therefore be important in the evaluation of HRQoL in thoracic sarcoma patients.

Tightness in the back and restrictions in performing tasks above arm height were location-specific physical issues for breast sarcoma patients. Restrictions in performing tasks above arm height are also known to be common issues in breast cancer patients. In the study of Blackburn et al. [36], breast cancer patients were interviewed who underwent a latissimus dorsi (LD) breast reconstruction after a mastectomy for breast cancer. They reported lymphedema, problems with wound healing, tightness in the shoulder and back, pain and discomfort at the donor site, reduced power, numbness, struggles with personal care (e.g., washing behind their back), and struggles with ADL (e.g., housework), which were also mentioned by the breast sarcoma patients in our study. Detection of early signs of physical impairments might be important in order to prescribe early interventions (e.g., physiotherapy) to optimally recover and return to presurgery levels of function. In the study of Blackburn et al., it also appeared that there was a lack of mental preparedness regarding the musculoskeletal impact of LD flap surgery. Research in other areas of surgery demonstrated that unrecognised or unfulfilled expectations are more closely associated with dissatisfaction than the technical success of the surgery [37]. In addition, patients who reported having fulfilled expectations had significantly greater gains in HRQoL than those who did not [38]. In patients undergoing breast reconstruction, understanding and managing patients’ expectations might improve patient satisfaction [39].

Body image issues and fear of recurrence (FCR) were often mentioned in both subgroups as major mental issues affecting their quality of life. Changes in body image are an important mental issue in breast cancer patients as well [40,41]. Breast surgery with partial or complete loss of one or both breasts can result in poorly aligned breasts and breast asymmetry, extensive scars, an alteration in the sensation of the breast and/or nipple, the need for a breast prosthesis, possible changes to arm mobility, and lymphedema [42]. Additionally, thoracic sarcoma patients experienced body image issues as a result of scars or holes after their surgeries, causing patients to experience restrictions in social activities. Issues of body image are often addressed after the completion of curative treatment. However, focusing on changes in body image before the start of treatment might prepare patients for certain outcomes after treatment. The significant FCR mentioned by the sarcoma patients in our study was provoked by the fact that patients were explicitly made aware of the chance of recurrence, because recurrence of sarcomas is common after surgical resection, in particular when adequate negative margins could not be achieved [43]. In addition, especially for thoracic sarcomas, surgery often leads to challenges with soft tissue coverage, with or without bony chest wall reconstruction, which can complicate subsequent surgical options if recurrence occurs [43] and also contributed to high FCR in our sarcoma population.

Taking into consideration the issues of thoracic and breast sarcoma patients, several g suggestions can be made for the development of a measurement strategy for sarcoma patients. The cancer-generic issues are already covered by existing cancer-generic HRQoL questionnaires (for example, the Functional Assessment of Cancer Therapy–General (FACT-G) or the EORTC-QLQ-C30). Nevertheless, it might also be important to use a specific questionnaire with more detailed questions on some specific issues, for example, by incorporating a short form of the brief pain inventory (BPI)) to assess type, location, and frequency of pain. In addition, specific questions on sleep disturbances can be added for example from the PROMIS sleep disturbance item bank in order to assess its cause. Fear of recurrence is an important issue in the sarcoma population and is underrepresented in current HRQoL questionnaires; therefore, it might be useful to add one or more items on FCR or an FCR-specific measure (e.g., FCR-I) in the specific questionnaire for sarcoma patients. Location-specific issues mentioned by thoracic and breast sarcoma patients were similar to issues experienced by patients with lung cancer (respiratory problems) or breast cancer (restricted arm mobility, tight feeling in the back), respectively. For the development of an item list (consisting of items available in an item bank), it might be considered to use (parts of) questionnaires that are already being used to measure HRQoL in lung cancer patients and breast cancer patients. For instance, (parts of) the EORTC Quality of Life Questionnaire Lung Cancer-13 (QLQ-LC13) or the Functional Assessment of Cancer Therapy-Lung (FACT-L) can be used for the item list for thoracic sarcomas and (parts of) the EORTC Breast Cancer-Specific Quality of Life Questionnaire (QLQ-BR23) or the Functional Assessment of Cancer Therapy-Breast (FACT-B) for the item list for breast sarcomas.

This study is the first to report on specific HRQoL issues in an international cohort of thoracic and breast sarcoma patients. The results of this study will guide personalised supportive care for breast and thoracic sarcoma patients. The in-depth analysis of thoracic and breast sarcoma patients’ experiences will contribute to awareness among health care professionals and the possibility to use this information in the consultation room which will be helpful for informed decision making. In addition, our study can help in determining the best possible measurement strategy for sarcoma patients with different primary tumour locations for research and clinical practice. This predefined subgroup analysis was part of a larger multicentre EORTC study that included patients with other sarcoma locations. All HRQoL issues that were reported in the interviews, will be consolidated into a comprehensive list of issues for the second part of the study by asking patients and HCPs to rate the HRQoL issues on relevance and priority and to indicate relevant issues missing from this list. This data will provide the possibility to determine subgroup-specific issues using a comparative approach. Our study also has some limitations. A larger sample size, especially for breast sarcomas, would have given us greater certainty that we identified all the key issues and data saturation had been fully reached. Nevertheless, this is a qualitative study, and the aim was not generalisability. Another limitation is the limited number of patients in the palliative phase; further subthemes could have emerged when they were included. Finally, positive issues (i.e., enjoying little things more when feeling good), coping issues, and issues related to the diagnostic trajectory were excluded, as they represent different concepts not incorporated in the HRQoL assessment.

## 5. Conclusions

Analysis of problems reported by thoracic and breast sarcoma patients revealed a wide variety of physical, mental, and social health issues, which can significantly impact thoracic and breast sarcoma patients’ HRQoL. Some of the issues were cancer generic (e.g., fatigue), but others were specifically related to the local impact of the tumour or treatment. Most location-specific issues in thoracic and breast sarcoma patients were similar to issues experienced by patients with lung cancer and breast cancer, respectively. Body image issues and fear of recurrence were also important challenges in both subgroups influencing HRQoL. Results of this qualitative study will guide personalised supportive care for breast and thoracic sarcoma patients and help in determining the best possible HRQoL measurement strategy for sarcoma patients with different primary sarcoma locations.

## Figures and Tables

**Table 1:** Patient characteristics.

	Thoracic (19)	Breast (4)
**Gender** *N* (%)		
Male	8 (42%)	0 (0%)
Female	11 (58%)	4 (100%)
**Age completing questionnaire** *Mean* ± *SD*		
(In years)	56 (13)	60 (19)
**Time since diagnosis** *Mean* ± *SD*		
(In months)	43 (37)	53 (22)
**Country of inclusion**		
Germany	5	1
Italy	4	0
Cyprus	1	0
Norway	1	0
United Kingdom	1	0
The Netherlands	7	3
**Marital stage** *N* (%)		
Single	5 (26%)	1 (25%)
Married	11 (58%)	1 (25%)
Living with partner	2 (11%)	1 (25%)
Divorced/separated	1 (5%)	0 (0%)
Widowed	0 (0%)	1 (25%)
**Living arrangements** * *N* (%)		
Alone	2 (11%)	2 (50%)
With partner	13 (68%)	2 (50%)
With children < 18	1 (5%)	0 (0%)
With children > 18	3 (16%)	0 (0%)
With parents	3 (16%)	0 (0%)
**Highest education** *N* (%)		
Primary school only	3 (16%)	0 (0%)
High school	6 (32%)	0 (0%)
College/university	9 (47%)	3 (75%)
Missing	1 (5%)	1 (25%)
**Employment** *N* (%) **		
Fulltime	4 (21%)	0 (0%)
Parttime	3 (16%)	1 (25%)
Unemployed	1 (5%)	0 (0%)
Homemaker	1 (5%)	0 (0%)
Retired	5 (26%)	3 (75%)
Disabled	3 (16%)	0 (0%)
Sick leave	3 (16%)	0 (0%)
**Sarcoma subtype** *N* (%)		
Soft tissue sarcoma	15 (79%)	4 (100%)
Bone sarcoma	4 (21%)	0 (0%)
**Sarcoma histology** *N* (%)		
Chondrosarcoma	2 (10.5%)	0 (0%)
Ewing’s sarcoma	2 (10.5%)	0 (0%)
Liposarcoma	4 (21%)	0 (0%)
Myxofibrosarcoma	2 (10.5%)	0 (0%)
Osteosarcoma	1 (5%)	0 (0%)
Rhabdomyosarcoma	2 (10.5%)	0 (0%)
Sarcoma NOS	3 (16%)	1 (25%)
Angiosarcoma ***	0 (0%)	3 (75%)
Other ****	3 (16%)	0 (0%)
**Stage at diagnosis** *N* (%)		
Localized disease	17 (90%)	3 (75%)
Metastatic disease	2 (10%)	1 (25%)
**Treatment at diagnosis** *N* (%)		
Chemotherapy	8 (42%)	2 (50%)
Radiotherapy	7 (37%)	1 (25%)
Surgery	17 (90%)	4 (100%)
Targeted therapy	1 (5%)	0 (0%)
Hyperthermia	1 (5%)	0 (0%)
**Treatment goal at diagnosis** *N* (%)		
Curative	17 (89.5%)	4 (100%)
Palliative	2 (10.5%)	0 (0%)
**Stage at study enrolment** *N* (%)		
Localized disease	3 (16%)	0 (0%)
Metastatic disease	3 (16%)	1 (25%)
Local relapse	3 (16%)	0 (0%)
Follow up	10 (52%)	3 (75%)
**Number of metastasis at study enrolment** *N* (%)		
0	15 (79%)	3 (75%)
1–3	0 (0%)	0 (0%)
4 or more	3 (16%)	1 (25%)
Unsure	1 (5%)	0 (0%)

* Sometimes multiple answers, so both with partner and with children <18 years or with partner and with children >18 years. ** One patient mentioned being disabled, but still worked part-time instead of full time. *** 1 Primary, 2 post-radiation. **** Follicular dendritic cell sarcoma, spindle cell sarcoma, pleomorphic sarcoma.

## Data Availability

After the study [7] is completed, the data will be stored in the EORTC Quality of Life Group Data Repository.

## Supplementary Materials

The following are available online at https://www.mdpi.com/article/10.3390/jcm10225334/s1, Table S1: Physical health quotes—thoracic sarcomas, Table S2: Mental health quotes—thoracic sarcomas, Table S3: Social health quotes—thoracic sarcomas, Table S4: Physical health quotes—breast sarcomas, Table S5: Mental health quotes—breast sarcomas, Table S6: Social health quotes—breast sarcomas.
