# Supplementary material for: Health-Related Quality of Life Issues Experienced by Thoracic and Breast Sarcoma Patients: A Rare and Understudied Group
Source: J Clin Med. 2021 Nov 16;10(22):5334. doi: 10.3390/jcm10225334 (PMC8618823; doi:10.3390/jcm10225334)
Supplement: Supplementary file 1 [file jcm-10-05334-s001.zip › jcm-1392567-supplementary.pdf]

**Supplementary: Quotes**

Table S1: Physical health quotes - thoracic sarcomas

| **Physical Health – Thoracic Sarcomas** |
|---|
| **Subtheme 1: Lack of Energy** |
| **Category 1: (Easily) tired** |
| Physically, I can do everything but I'm just tired faster. I can't do many things because I get tired quickly, that's the only problem I have. I just do what I can. [Patient 1] |
| Workwise I now work part time because I get tired easily. [Patient 3] |
| **Category 2: Feeling weak and lacking energy** |
| I just wanted to sleep, because after the second chemo I had anaemia. I had no strength at all. [Patient 5] |
| I had to sit while taking a shower. [Patient 4] |
| I go swimming once a week. I started doing that again, so that's progress. I'm not as good as I used to be. I don't have the same energy as I used to have. [Patient 6] |
| I often feel like taking a nap on the couch. [Patient 7] |
| At a certain point, if I was sitting in the living area and I wanted to go outside, I had to sit on a chair halfway through the room near the dining area, and also in the kitchen, because I was so weak. [Patient 4] |
| **Category 3: Need to rest** |
| At a party no one noticed anything, but they didn't know I had slept at home from six to nine. [Patient 4] |
| **Subtheme 2: Sleeping problems** |
| I have stress and difficulty sleeping if I can't do something. [Patient 8] |
| I think that my breathing has changed a bit. I just have to take a very deep breath every now and then. It is as if I am not getting enough oxygen. I'm a little bit scared about that or panicky sometimes. It is annoying. See, normally during the day I have no problems with it, nor do I suffer from it. But when I am in bed, I am paying attention to it in a different way. [Patient 10] |
| **Subtheme 3: Pain** |
| The most important thing I experience is pain. [Patient 3] |
| I experience some changes in my normal daily activities, mostly due to prolonged fatigue and sometimes pain. [Patient 11] |
| I experience pain when lifting heavy weights. [Patient 8] |
| After the surgery I had a lot of pain, I could not sit or lay down, but now it is not a problem any more. [Patient 12] |
| **Subtheme 4: Respiratory problems** |
| **Category 1: Coughing** |
| I cough which limits my conversations. [Patient 13] |
| **Category 2: Breathing problems** |
| I just have to take a very deep breath every now and then. It's like I am not getting enough oxygen, like I just can't get enough breath through my nose or like my nose is actually closed. [Patient 10] |
| I have problems with breathing. [Patient 14] |
| I am short of breath. [Patient 15] |
| **Category 3: Voice problems** |
| My voice is affected, I can only whisper. [Patient 13] |
| **Subtheme 5: Chemotherapy related problems** |
| **Category 1: Mouth problems** |
| Chemotherapy caused mucositis, this caused swelling in my mouth, I could not open my mouth at all. [Patient 12] |
| I had wounds in my mouth. [Patient 16] |
| **Category 2: Feeling ill** |
| At one point I had three more chemotherapy sessions, and it felt like I had to climb the Alpe d'Huez three more times. It felt that way. The total decay, that was tough. [Patient 4] |

| |
|---|
| The first few times I had a lot of complaints. I had to vomit constantly and I was very sick. The first two days went well but after the third and fourth day I had little appetite and it just made me feel bad. [Patient 16] |
| The chemo cycles were too short after each other, I needed a longer period in between. [Patient 12] |
| I also felt more and more miserable, when I went home again I usually had another week in which I felt bad, but after that I recovered, and I thought, “Do I have another good week?”, but I already had to go again. And that was fourteen times, it was a lot and a very long time. [Patient 4] |
| **Category 3: Changes in consciousness** |
| I got hallucinations. Sometimes I heard things that others did not. I had that when I closed my eyes, I went to a place with nothing but flowers. Beautiful flowers, sunflowers, roses, really beautiful! And when I opened my eyes again, I realized where I was. [Patient 5] |
| **Category 4: Nausea/vomiting** |
| I was occasionally nauseous, but it was not continuous. [Patient 5] |
| I really had to vomit constantly and I was very sick. [Patient 16] |
| **Category 5: Hair loss** |
| My hair started to fall out a bit. [Patient 5]. |
| **Category 6: Lower resistance to infection** |
| I didn't go to birthdays because it made me sick, because at a certain point my resistance became worse and worse [Patient 16] |
| **Category 7: Eating problems** |
| Less appetite, little taste. All food tasted different. Normally I am a very good eater, but then I had less appetite. [Patient 16] |
| I could not chew, not eat. I was not hungry and not thirsty. I had pain while eating and drinking. [Patient 12] |
| Furthermore, I did not want to eat much, I was not hungry. [Patient 5] |
| **Subtheme 6: Radiotherapy related problems** |
| The only side effect really was my skin. [Patient 6] |
| Well, my oesophagus got hit and at a certain point I couldn't eat anymore, I needed a feeding tube for a few months. [Patient 4] |
| **Subtheme 7: Surgery related problems** |
| **Category 1: Direct** |
| **Subcategory 1: Wound problems** |
| For a moment it was kind of infected. [Patient 7] |
| But it was in a very difficult place and I don't have that much fat and skin. So they took a muscle from behind and brought it forward and removed a slice of skin from the leg and placed it on my back. That looked nice in the beginning, but after two or three days it went downhill. The blood flow was not good, so the wound had to be opened every time, every two hours or so, even at night. And it all infected. It was very dirty. They operated me to clean everything properly. I had to lie down for two days and then they placed a vacuum pump on it. Well that was not nice because the vacuum pump started to beep every now and then. [Patient 10] |
| **Subcategory 2: Lymphedema** |
| I’m having lymphedema now in my left arm. [Patient 6] |
| **Category 2: Indirect** |
| **Subcategory 1: Area of surgery is bothering / feels different** |
| I notice that I have some back problems. I've always had a bit of a nagging spot at the place where I had surgery. But that's just an inconvenience. So it’s not that I can't do things because of it. And at the left side of my chest, in the middle, I don’t feel as much as I used to. That feeling has not returned. It feels like a weird tingling or a numb feeling. But that is also an inconvenience. [Patient 16] |
| The doctor has removed two ribs. He removed the bottom floating ribs and a mat has been placed in it. Now and then I still suffer from that mat. When I turn around, I can feel it stinging a little. [Patient 2] |
| Just that whole area is a drama. It's just one big, hard lump. So when I do something with my arm, it's like… Well, imagine you're wearing a three-quarter sleeve t-shirt from your six-year-old nephew. So every movement you do, it works, but it feels like it’s impossible. And that’s how it feels for me too. So I just can’t |

| |
|---|
| say it hurts, but it just feels like you're making a move that you actually can't. Like you're overstretching your arm. [Patient 7] |
| **Subtheme 8: (Functional) impairments** |
| **Category 1: Physical impairments** |
| If I have to climb a mountain during the holidays, I have some problems. I don't have that endurance. I will come to the top, but it takes a little longer. We used to walk together, but now my wife is faster. In the past we walked fairly equal and that is no longer the case. [Patient 10] |
| Yes, I have always walked long distances. But eventually we just had to shorten it, just through the village. I kept walking, at least until the end, then it was no longer possible. [Patient 4] |
| I just can't finish things. If I have a bad day, then I have projects all over the house that have finished halfway. That irritates me. [Patient 7] |
| I am no longer totally able to do anything I did before. I can still do some activities, but not all of them. [Patient 11] |
| I can't bicycle, I have difficulties driving and walking long distances [Patient 14] |
| **Category 2: Impairments in ADL** |
| I experience physical constraints when doing household chores and some daily activities. [Patient 17] |
| Vacuuming, making beds and things like that are hard for me. I can do it now, but back then my husband took over a lot [Patient 2]. |
| I have difficulties with heavy work. [Patient 8] |
| **Category 3: Impairments in mobility** |
| I sometimes have an ache in my arm where my lymph nodes were removed, it's a bit stiff but it's not pain. [Patient 6] |
| I had some problems with my shoulder. You can feel it, there is a lump and it bothers me that I just can't put my arm right next to my body, which causes me to walk differently than normal. And if I keep that up long enough, it gets a bit stiff. That has been loosened by the physiotherapist so now I can move my shoulder a bit more. [Patient 10] |

Table S2: Mental health quotes - thoracic sarcomas

| **Mental Health – Thoracic Sarcomas** |
|---|
| **Subtheme 1: Worries / fear** |
| **Category 1: Changed breathing** |
| I just have to take a very deep breath every now and then. It is as if I am not getting enough oxygen. I'm a little bit scared about that or panicky sometimes. [Patient 10] |
| **Category 2: Recurrence / disease progression** |
| It is the progression of the disease that most worries me, as changes in symptoms or other vital factors are increasingly frequent. These changes have a major impact on my quality of life, which is not favourable. [Patient 11] |
| I often feel very anxious, particularly about my health situation. I always feel anxious near the follow-up visits. [Patient 9] |
| When the result are good again, I am euphoric, but that mostly only lasts a month, and then I realize that I have another scan in three months. And by the time of the scan, I am really tense. The scan is something that just has to be done, and the tension comes usually anyway, mostly about one or two days before the results. And especially the night before. [Patient 4] |
| **Category 3: Death** |
| The moment I was diagnosed with sarcoma, and it was not entirely clear whether I had metastasis or not, I had total panic. I thought: gosh, actually I still have to do a lot of things... Yes, I was prepared for the cancer to come back, but when I heard that it was a sarcoma and that there was also a chance that it would just suddenly end within a few months, I thought: I am not prepared for that. I think that is really intense about this beast. That it was clear that there was also a possibility that it was just over and out very quickly. [Patient 7] |

| |
|---|
| At night when you are awake, you start to worry because you are already arranging your funeral in your head, you always think the worst. [Patient 2] |
| **Category 4: Work / financial** |
| I worry about money, I am trying to calculate at the beginning of the month what I need and I get financial support from my girlfriend. [Patient 18] |
| **Subtheme 2: Living with uncertainty** |
| I never felt cured either, it is known that it is a tumour that can come back quickly. [Patient 4] |
| The partner of a friend of mine, they discovered the cancer almost at the same time as mine. She was buried the moment I had my first check-up after the operation. So it was discovered almost at the same time and she is no longer alive and I'm sitting here telling my story now, I still find that difficult. So the tumour could just explode and they'll send you home and say, "Well, there's nothing we can do anymore". Yes that is what makes it mentally difficult for me. [Patient 7] |
| No, fear is too big a word, but I don't feel completely reassured that it is good now. The fact that the scan does not show anything, means that at least there is nothing larger than 2 millimetres, but I am not yet confident that those bugs, if they are there, will keep quiet [Patient 7] |
| In the beginning I was really tense for the results. The first year for sure, because there were some checks where they saw something, and the doctors were like, well we don't know what it is, so we suspect, they literally said that, we suspect it's back. And afterwards it turned out to be a blood clot or inflammation from the operation. So in the beginning, that uncertainty made it a bit difficult. [Patient 16] |
| Yes, in the beginning I found it very difficult, the uncertainty, because in the beginning they did not know exactly what it was and what my trajectory was going to be. [Patient 16] |
| The diagnosis of sarcoma is emotional... Just because it is such a false beast. With the breast cancer, I still had the false safety of: well, it might go wrong, but it will take a while. I still have some time. And with this one it feels like it is panting in my neck. It really feels like a race against time. Next month it could be all wrong and be done. Now and then that bubbles up, that realization. I have to deal with that. [Patient 7] |
| **Subtheme 3: Changes in personality** |
| I cannot keep my temper as I used to do. I am constantly on the edge. [Patient 3] |
| After the first diagnosis I was very aggressive and grumpy but this decreased through conversations with my psychologist. [Patient 18] |
| Well I am a primary school teacher. And when you've actually been out for a year, you question yourself, can I still do it? You have lost a bit of confidence in yourself. Not only in your body, but also in general. And that was in the beginning just like with those checks, by doing it, it all came back. [Patient 16] |
| **Subtheme 4: Changes in emotions** |
| I experience changes in my personal feelings, I often feel depressed. [Patient 11] |
| Well I think my traits are a bit different. I became emotionally different, I think I am a bit more sensitive. [Patient 10] |
| **Subtheme 5: Changes in cognitive function** |
| I sometimes have concentration disorders. [Patient 11] |
| **Subtheme 6: Body image** |
| It was during the first chemo, I looked at myself in the bathroom mirror and thought I am very ugly. My hair started to fall out a bit. [Patient 5]. |
| Interviewer: “Do you have difficulties with how it looks after surgery”<br>Patient: “Yes, it’s not pretty, but I don’t want anything, no surgery. Only when necessary, but not aesthetically. I discussed this theme with my sister. If I was thirty or twenty-five it was different, but I'm fifty-five. After surgery, I went to the beach once, but I had nothing with me to cover it. I still don't like wearing spaghetti straps in the summer either.” [Patient 5] |
| I find it difficult to go to the sauna because I don't want other people to stare at me. I'm not going to the sauna anymore because I can't pretend I don't see the people looking and I can’t pretend that it doesn't affect me, because it does affect me. I just find it annoying when people stare at me. I come there for relaxation, not to get stressed up because people are looking at me. [Patient 7] |
| No, it really doesn't look pretty. I can be honest about that. It really doesn't look pretty. Over time it has become tighter and tighter, the scar. With some women in the picture I see such a really beautiful stripe like that, and |

| |
|---|
| then nothing else. With me, because I've had so many complications with surgery, after surgery with infections, I just have some really weird holes and spots, and there is now also a pacemaker in it, and the Port-a-Cath used to be in there too. So that's one big war zone. But it doesn't affect me, but I notice that it affects other people when they just see it for the first time [Patient 7] |
| It doesn't look pretty either, of course. The second time has been a huge operation, the nipple has been removed from there. So it doesn't look nice and sleek anymore. [Patient 10] |
| Because socially, I can't go to a party, to a friend's birthday. I cannot go without hair, I didn't use a wig. I think it is so ugly [Patient 5] |
| **Subtheme 7: Normal life** |
| I play the trombone, but that was no longer possible. In the beginning I continued to do that every time in my third good week. But that became heavier, and in the end it was not possible. Then I still had a period when I still went to rehearsal, to the last part, there was usually a drink. But yes, over time it all became less of course. And then my husband would take me there by car if I wanted to. You have to consider, it's in only six, seven hundred meters, the rehearsal room, but he would take me by car, and he would pick me up again. I wanted to keep doing that because I just wanted to live. Because I've always been very social and that's just part of my life for me. I continued to do that, and I subconsciously think, maybe I did that, because I wanted to feel normal. [Patient 4] |
| Every once in a while when people come to my work and they say, oh why did you cut your hair short? I say: I wanted it different. If I feel like I don't want to say it, I say well it was dry and I wanted something else. [Patient 5] |
| Only during the hospital stays, afterwards I tried to go back to daily life as soon as possible [Patient 18] |

Table S3: Social health quotes - thoracic sarcomas

| **Social Health – Thoracic Sarcomas** |
|---|
| **Subtheme 1: Job** |
| **Category 1: Not being able to work** |
| I am partially disabled, because of concentration problems and fatigue complaints. [Patient 4] |
| I can't do my job anymore. The problem is that I can't lift heavy things and I used to work as a mason [Patient 12] |
| Workwise I now work part time because I get tired easily and have bone-related pain [Patient 3] |
| **Category 2: Adjustments needed to work** |
| I was lucky, because I worked twenty-one hours, of which I worked five mornings at the municipal. In the morning I made appointments with clients, and then I went home to sleep. At home I logged in again, and I reported everything. And well, luckily I have such a job that that was possible. Because as a nurse you have to be at the bedside of your patient. Then you cannot work from home. [Patient 4] |
| The stuff has to be unpacked, I also had to be at the top of shelves. That is not possible anymore. So I sometimes have help when a lot of stuff comes in. [Patient 10] |
| For example, my surgery is planned in December. So we had a meeting last night, and I said: "I will hopefully have an operation in December, but I don't know exactly when. I also don't know how long I need recovery time. That means I can't actually schedule appointments that can't be cancelled last minute. So I can't give a lecture or something, because it is almost impossible to cancel that. [Patient 7] |
| **Category 3: Missing work** |
| Well I am a primary school teacher. I had been actually out for a year, and I had the feeling that I had missed a lot in the first place. [Patient 16] |
| **Subtheme 2: Financial** |
| **Category 1: Insurance** |
| The life insurance became much more expensive. Also if you would like to move and your mortgage needs to be converted, than a lot more work is needed than if you are healthy. [Patient 16] |
| **Category 2: Health care costs** |

| |
|---|
| I said to my husband that I would like to get some help in the household. But that is also a financial loss of course so then you start to think, is that the most important thing? [Patient 2] |
| And a wig, it was expensive too, nine hundred euros. Yes financial… I have saved, but everything is going away now. Because you also have to pay your own risk. [Patient 5] |
| **Category 3: Financial difficulties** |
| I only received 280 euros a month, that's not enough money for my disability. [Patient 8] |
| I get financial support from my girlfriend, because I am disabled, so I can't work anymore. [Patient 18] |
| **Subtheme 3: Loss of independence** |
| My husband stayed home from work at that time, so he took care of the children along with my parents and his parents. [Patient 16] |
| And friends of mine, for example, cleaned the house, because it had to be very clean due to the risk of infection. [Patient 16] |
| I found it annoying to depend on others. [Patient 14] |
| Family and friends helped very much in the first few month [Patient 14] |
| **Subtheme 4: Relationships** |
| **Category 1: Changes in relationships** |
| Sometimes I struggled with my mother's coping style, it is different than my own, so occasionally that led to discussions. [Patient 18] |
| I am still meeting my friends but what changed is the content of our conversations from their side. There is sometimes too much caution about me and what I can do and sometimes they suggest things to do that I cannot do and that makes them feel embarrassed. [Patient 3] |
| Relationships with friends have not really changed, except that it is not as regular as before due to my restricted mobility and fatigue. [Patient 11] |
| **Category 2: Feeling guilty and don't want to be a burden** |
| For my wife it was of course quite a shock, because a lot came down to her in that period [Patient 10] |
| I found it difficult to tell the news to my family. Because I felt a little guilty. They start crying and ask why?, nobody knows why, so I felt a little guilty for hurting them. [Patient 5] |
| And now the last time our son came with his girlfriend, and they said they wanted to get married, on my birthday. So he said, "You will probably never see us having children and if we get married now, you can still be there". I said, "You completely ignore your own wishes." But yesterday they explained, sometimes wishes change, and that is now. Well, and of course I spoke to my sons mother-in-law, who also said: "Listen, my daughter never does anything impulsive". That makes you feel guilty, doesn't it? [Patient 4] |
| And you always have a tendency to protect them a bit. I am honest, they also want me to be honest, but I don't always show the back of my tongue and as long as I'm fine, I actually want them to just live their lives. [Patient 4] |
| **Category 3: Isolation** |
| You definitely notice that it has some impact and your world gets a little smaller. [Patient 16] |
| I felt isolated right after the diagnosis [Patient 9] |
| **Category 4: Lack of understanding / criticism** |
| I have less contact with my grandmother due to her instructive behaviour towards me and I also struggled with my mother's coping style, it is different than my own, so occasionally that led to discussions.. [Patient 18] |
| And that is what I mean with people, they sympathize, but they cannot empathize if you do not experience it yourself. [Patient 4] |
| Some people said to me: "How nice, that you are cured again." And in the beginning I once explained, "Well, I am not cured, it is known that it is a tumour that can come back quickly. Actually, we just have to wait and see when it will come back". I once found that difficult. And if I explained it, they still didn't understand that. I think they saw what they wanted to see. [Patient 4] |
| In the beginning they first thought in the direction of lymph node cancer. In that week that they thought I had lymph node cancer, I already got a lot of well-intentioned advice, people who googled. When it became clear that it was something else, I thought, I don't need all that well-intentioned advice. If they don't know what I have, they can't google it. And I have maintained that so far. And even now, my kids don't know what sarcoma I have. |

| |
|---|
| I've always said, "Just a tumour." But not the type. So they couldn't google what the prospects were. I protected myself against it. Not because they weren't supposed to know, but all that well-intentioned advice from people who google, I don’t want that. My husband and I said if I want information about a piece of meat I will go to the butcher, and if I want information about my tumour I will go to the hospital. I don't search the internet myself and others don't have to do that for me either. And it is well-meaning, but I found that very annoying. So I have protected myself against that. [Patient 4] |
| My wife and my kids have had a lot more trouble with the diagnosis than I had.<br>Interviewer: “And does that affect you?”<br>Yeah, it's not pleasant, but I told them, I don't mind, so then you shouldn't mind either. That also makes it a bit easier for me. [Patient 1] |
| **Subtheme 5: Limitations in social activities** |
| I cannot have a busy program in the morning, and have a busy program in the afternoon, and in the evening. So it's either one of them. Last Friday someone had tickets for a concert and it was great fun. But I already knew in advance, because on Saturday I also had to teach, that on Sunday I would just lie sick and tired on the couch. So more often, I cancel social things, or I postpone them, because I know the price I have to pay. [Patient 7] |
| I have to plan social activities according to the other activities I have to do because of tiredness [Patient 14] |
| I cannot do sports the way I used to or do gardening which I used to enjoy, because I get tired easily and I have bone-related pain. [Patient 3] |
| I play the trombone. Yes, of course that was no longer possible. [Patient 4] |
| I go out less, walking was a former hobby of me, but sport is impossible at the moment [Patient 13] |
| I can't play football anymore because I can't run due to my missing ribs [Patient 12] |
| I didn't go to birthdays because I couldn't handle the crowds very well and I was afraid that it would made me sick or I would feel sick, because my resistance became less and less. So yes, I spent a lot of time with my own family and close friends. So the world suddenly became a lot smaller. And that is on the one hand very nice, because then you grow closer together. But on the other hand, you miss a lot. [Patient 16] |
| I just can't do some hobbies. I love to be in the garden, but after ten minutes I have to sit down and that irritates me. [Patient 7] |

Table S4: Physical health quotes - breast sarcomas

| **Physical Health – Breast Sarcomas** |
|---|
| **Subtheme 1: Lack of Energy** |
| **Category 1: (Easily) tired** |
| I have to say that I am very tired. I didn't notice that during the chemotherapy, but I notice it a lot more now. After cooking, I am exhausted, I have to lie on the couch and I fall asleep. [Patient 21] |
| **Category 2: Lacking energy** |
| I have very little energy and I don't recognize that off myself. [Patient 20] |
| My energy has decreased a lot [Patient 21] |
| **Subtheme 2: Sleeping problems** |
| I was always a very carefree lady and very cheerful and happy and now I have actually become a terrible worrier and that affects my sleep. [Patient 20] |
| All kinds of things are going through my head and that affects my sleep. I sometimes wake up at 3:00 am and think how will my life continue and I just can’t fall asleep. And that’s probably because I can’t share it with a partner because I live alone. [Patient 21] |
| My position in bed is also a bit difficult, because I cannot really lie on my back or my side. So sometimes I wake up at night and then I am really stiff and I almost can't get out of bed. [Patient 20] |
| **Subtheme 3: Chemotherapy related problems** |
| I also had a lot of heart palpitations during the chemotherapy [Patient 21] |
| When I walk in the hills near my house, I am short of breath. It is especially during exercise and also when I climb the stairs, I never had that before. [Patient 21] |
| I pee a lot more and I am less able to hold it. Since the chemo, I wear a diaper. [Patient 21] |

| |
|---|
| I have been really ill. As if you have the flu, with a really bad headache and vomiting. And when I just recovered a bit, I had to go again and I found that very difficult. [Patient 20] |
| What I still often have, is that food comes up from my stomach. [Patient 21] |
| My toenails are coming off [Patient 22] |
| I have sagging legs. [Patient 22] |
| **Subtheme 4: Radiotherapy related problems** |
| From the start of the first radiation treatment, I am short of breath. And last year I already had 22 radiation treatments and chemotherapy, but it has not improved [Patient 20]. |
| **Subtheme 5: Surgery related problems** |
| **Category 1: Direct** |
| **Subcategory 1: Wound problems** |
| I had a major operation and that is very nicely done by the plastic surgeon. Later I had an infection of the wound, so I went back to the hospital a number of times and they opened it again and I had to flush it in the morning and evening. [Patient 20] |
| I also had a pump here, because I had a very big hole, the wound closed very difficult, and it had to be flushed. [Patient 22] |
| **Category 2: Indirect** |
| **Subcategory 2: Indirect problems related to surgery** |
| When I have to go upstairs to sleep, I have to do that very carefully and hold the handrail. I feel the muscle that they have brought forward from my back, I feel that muscle protesting and that causes shortness of breath sometimes. But I learned from the physiotherapist how to bring back my breathing to normal. [Patient 20] |
| They also brought a part of my back muscle to the front and for the back they had nothing to put in between so they pulled that together and I constantly have a very tight feeling there because that is of course very tight, it feels like a car tire. In the evening, my back really hurts. [Patient 20] |
| They took a muscle out of my back and put it here. It bothers me terribly. If I get nervous or something else, it pulls very tight. And sometimes I think, oh, I still have my bra on, but I've already taken it off. It feels that tight. [Patient 22] |
| **Subtheme 6: (Functional) impairments** |
| **Category 1: Physical impairments** |
| I experience restrictions with increased physical activity, like for example sprinting and I have difficulties with climbing stairs, especially more than three floors. [Patient 23] |
| Overall I am limited and I have to live with that [Patient 20] |
| **Category 2: Impairments in ADL** |
| I can shower and dress myself, that is no problem. But I have problems with doing house hold chores, heavy household chores. The light work is no problem, as long as it is on arm height. I've already had a recovery operation so now I can move my arm a little higher, but that's not the way it was. What I find most difficult is cleaning on top of cupboards or cleaning windows. [0a1-009] |

Table S5: Mental health quotes - breast sarcomas

| **Mental Health – Breast Sarcomas** |
|---|
| **Subtheme 1: Worries / fear** |
| **Category 1: Recurrence / disease progression** |
| If I feel something strange, I am always scared. You live in fear. Also because it is so rare. Breast cancer is of course also very bad, but there are many more positive results and there are more people who get better. [Patient 21] |
| I am very afraid because the oncologist in the hospital said, “it is an aggressive form and it is like weed, it can come back anywhere any time. That does affect a whole part of my life. I think that's the biggest problem. The fear that it can come back every moment. [Patient 20] |
| **Subtheme 2: Living with uncertainty** |

| |
|---|
| I will get the results shortly and that is scary every time. Last time it had become a bit smaller, but yes, you have to wait every time and you are never sure if it will be back. And if that's the case you have to fight again, that's just the way it goes. [Patient 22] |
| **Subtheme 3: Changes in emotions** |
| I feel a bit down, next week I will have a conversation with a psychologist, my general practitioner thought that was important. [Patient 20] |
| I have actually become a terrifying worrier, I can't relax. That has changed my personality. Not only I think that, but the people around me also notice it. [Patient 20] |
| I'm emotionally more vulnerable. [Patient 23] |
| I am very tense and sad. [Patient 20] |
| **Subtheme 4: Changes in cognitive function** |
| I have difficulties concentrating and remembering things. My house is full of notes. [Patient 20] |
| I have difficulties remembering things. When I go upstairs, I forgot why I went. When I go down again, then I remember why I went upstairs. I heard that it could be caused by the chemotherapy. [Patient 21] |
| **Subtheme 5: Body Image** |
| I hated that I had no breast. I had seen it in pictures. With me there is a cut from here to there, even up to here. I hate it. I think it looks awful. At first I didn't look. Now I look when I am standing naked in front of the mirror. But in the beginning I could not look at it. [Patient 21] |
| I hardly dare to look in the mirror because now I am wearing my prosthesis again and I cannot really look at it properly. I personally think, if you ask me, I think it is a mutilation of my body. But I had no choice, I had to. [Patient 20] |
| I remember that I had shown it to a friend who was shocked. I am very open. I take off my shirt and I show it. But it is also difficult how to deal with the reaction of others. [Patient 21] |
| I cannot wear a low neckline, because of my breast amputation. [Patient 23] |
| My grandchildren don't see me without hair. I now have some hair back, but it is not much, I don't like that. They saw it once but they were a bit shocked. [Patient 22] |
| I did have that cooling on my head because I have small grandchildren and another grandmother had died a year before and she had a bald head and I thought I do not want that for my children. [Patient 20] |
| It was thinner of course, but you keep your identity and you stay yourself. I had looked up all kinds of things on the internet where you could buy a nice cap. I think they are horrible things first of all. I think it's like a bathing cap that you have on your head. I thought there must be something more fun, so I finally found a lady in Belgium who did something nicer with a bow at the back, so I also bought that. I thought if my hair falls out despite the cooling then I have at least those options. But a wig, I'm not going to put that on. I hate it, it's like chemical hair when you see it. I sometimes see someone with a wig and then I think it's terrible, I can't take my eyes off it. It was very important for me that I could just walk down the street like nothing is wrong with me. You couldn't tell that I had or have cancer. I could wear it on the streets, I really liked that. Very nice to have. [Patient 21] |
| I know that my breast is going to be amputated and that is a big problem for me. I find it quite confronting. I find those pictures that I saw on the internet very confronting [Patient 21] |

Table S6: Social health quotes - breast sarcomas

| **Social Health – Breast Sarcomas** |
|---|
| **Subtheme 1: Financial** |
| Financially, I really hated all the money I had to spent. You have to buy a bathing suit and it immediately costs € 140 because you have to put a prosthetic in. You have to buy a prosthesis. Clothing with a deep neckline is no longer possible, so I have to wear a shirt that is high. I still wear normal bras, but actually you also have to buy new bras, all those idiotic things are very expensive. [Patient 21] |
| **Subtheme 2: Loss of independence** |
| After those drains were removed, I was allowed to go home where I got help putting on my elastic stockings and help showering. I thought it was so bad that you were sitting naked on a bench in the shower, and other |

| |
|---|
| people had to shower me. I thought that was terrible. Anyway, it was good that they were there because I couldn't do it myself. [Patient 20] |
| I'm not going to tell the medical examiner about those neuropathic feet, because then I won't get my driver's license anymore and that is actually very important for me to drive independently from A to B. [Patient 21] |
| **Subtheme 3: Relationships** |
| **Category 1: Changes in relationships** |
| There was someone with me to the appointment and I asked the Dr. “can I arrange my will already”. Then the person who was with me to the appointment said “you are certainly not in your coffin yet”. That triggered me so much, I ended that contact, because that hurt me so much at that time. [Patient 21] |
| **Category 2: Don’t want to be a burden.** |
| My children are shocked when something happens. They have a hard time too. And I feel sorry that they are sad. [Patient 22] |
| I am alone, and I can't always get everything of my chest, because I don't want to burden my children with it every time [Patient 20] |
| **Category 3: Lack of understanding** |
| She didn't understand it at all. She just give me a call from “you're better”. I said: “where did you get that news from, mom?”. I said, “I am still under control and it can still come back and the Dr. has also said it's a 50-50 change that it will come back”. She didn’t understand that. I had to tell her that several times. It's just that those sarcomas are so unpredictable they can't say much about that. I had to tell her that a number of times. [Patient 21] |
| That is probably because I cannot share it with a partner because I live alone. And people also do not understand what it means and what it does to you to get such a message. [Patient 21] |
| You are the one who has the disease and you are also the one who also has to tell, it’s not like this, it is like this. That also has an impact on yourself. That’s hard sometimes. You also need energy for that. [Patient 21] |
| It was a difficult time, because, my father was dying, my mother had a little dementia and she did not understand everything so I had to explain it to her every time, that was very annoying. [Patient 22] |
| **Category 4: Isolation** |
| I try to get on my bicycle. I have bought an electric bicycle. But I always come home in an empty house, I find that difficult. [Patient 20] |
| **Subtheme 4: Limitations in social activities** |
| I was also crazy about working in my garden. That is no longer possible because I cannot get on my knees, so I now have a man who takes care of my garden. It sometimes makes me really rebellious that I can't do that anymore. Physically I just don't have the strength anymore. [Patient 20] |
| I like to run, but I can't do that anymore. They think that’s probably due to the chemo. I find that very annoying, that all of that is taken away. [Patient 22] |
